# Transient 2D Junction Temperature Distribution Measurement by Short Pulse Driving and Gated Integration with Ordinary CCD Camera

**DOI:** 10.3390/s22155899

**Published:** 2022-08-07

**Authors:** Zhiyun Wang, Honglin Gong, Peng Zhuang, Nuoyi Fu, Lihong Zhu, Zhong Chen, Yijun Lu

**Affiliations:** 1 Department of Electronic Science, Xiamen University, Xiamen 361005, China; 2 Xiamen Products Quality Supervision & Inspection Institute, Xiamen 361000, China

**Keywords:** thermoreflectance imaging, gated integration, transient temperature

## Abstract

The time resolution of the transient process is usually limited by the minimum exposure time of the high-speed camera. In this work, we proposed a method that can achieve nanosecond temporal resolution with an ordinary CCD camera by driving the LED under test with a periodic short-pulse signal and multiple-cycle superposition to obtain two-dimensional transient junction temperature distribution of the heating process. The temporal resolution is determined by the pulse width of the drive source. In the cooling process, the Boxcar gated integration principle is adopted to complete the two-dimensional transient junction temperature distribution with temporal resolution subject to the minimum exposure time of the CCD camera, i.e., 1 μs in this case. To demonstrate the validity of this method, we measured the two-dimensional transient junction temperature distribution of the blue LEDs according to the principle of thermoreflectance and compared it with the thermal imaging method.

## 1. Introduction

The light-emitting diode (LED), characterized by small size, high brightness efficiency, good luminous efficiency, fast response time, high reliability, and environmental friendliness, has been widely applied to various fields, e.g., general high-resolution display or sensing applications. Junction temperature has a significantly negative impact on the performance of LEDs, such as the luminous efficiency, stability, and service life [1,2,3]. Furthermore, in sensing applications, the junction temperature is critical where the high-power LED replaces the LD in the optrode pair [4] or fluorescence is excited by the LED [5]. It is important to control the junction temperature at a reasonable level to maintain the performance of the LED. A number of methods have been proposed to detect the LED thermal characteristics. The key point of existing junction temperature measurement methods usually lies in seeking the temperature-sensitive parameters (TSPs), including the temperature-sensitive electrical parameter (TSEP) and the temperature-sensitive optical parameter (TSOP) [6,7,8,9,10].

Conventional methods tend to treat the LED chip as a whole and evaluate the thermal performance of the chip by measuring the average junction temperature [11]. The thermal measurement technique has recently been extended to two-dimensional (2D) or three-dimensional (3D) junction temperature distribution. Kim et al. [12] came up with a confocal thermoreflectance microscope that employs auto-balanced detection for precise backside thermal fault localization. However, the intensity of the pseudo-thermoreflectance signal is relevant to the speed and range of scanning. As a consequence, the scan speed was not changed, and only the thermoreflectance images with 500 × 500 pixels in 50 s with a pixel dwell time of 100 μs could be detected. Gao et al. developed a contact measurement technique that moves a thermal probe discretely across a large-area bonded substrate and acquires the interface thermal resistance at each location. Chips with a size larger than 2 × 2 mm^2^ on the surface of the LED under test (LUT) could be detected at the cost of time accuracy [13]. Considering the practical application, noncontact measurement methods are more suitable for 2D thermal distribution measurement of LED to avoid risking the destruction of chips by probe contact [14,15,16]. Xiao et al. [17] proposed a method to measure 2D transient junction temperature distribution with a high-speed camera. The time resolution was subject to the temporal resolution of the camera itself, generally at the scale of 10–100 μs. Wang et al. developed an optical thermoreflectance imaging setup with 100 nm spatial resolution and 50 ns temporal resolution for dynamic temperature evolution under proper manipulation of probe pulses [18]. A high-resolution camera is usually the key instrument in the high-temporal-resolution measurement technique. Intensified charge-coupled device (ICCD) cameras and streak cameras are usually employed for high-speed imaging such as fluorescence lifetime imaging and combustion field imaging due to their ultrahigh temporal resolution [19,20]. Specifically, temporal resolutions in the femtosecond range can be achieved by streak cameras. However, the measurements are limited to one spatial dimension [21]. To construct and image a 2D scene, additional moving parts such as scanning elements are required to perform multiple measurements across the extra spatial dimension [22,23]. ICCD is able to achieve temporal resolution at nanosecond or even sub-nanosecond scale [24,25,26]. The ICCD and streak camera are expensive scientific instruments. One might expect that an ordinary camera could be competitive with the ICCD or the streak camera in terms of temporal resolution by applying new measurement principle.

A two-dimensional transient junction temperature distribution method, micro high-speed transient imaging based on reflected light (µ_HSTI), is proposed in this paper. Notably, the LUT is driven by a nanosecond periodic short-pulse signal, while an ordinary camera is adopted to obtain the 2D transient thermal reflection imaging with nanosecond-scale temporal resolution.

## 2. Theory

The relationship between the reflectivity and the temperature of the LED chip can be linearly expressed as follows [27]:

(1) $$R\left(T\right)\approx R\left(T_{0}\right)+\frac{\partial R}{\partial T}\left(T-T_{0}\right).$$

In practical terms, R(T) and $R\left(T_{0}\right)$ can be replaced by the relative intensity of reflected light of incident light *L*(*T*) and $L\left(T_{0}\right)$ at temperature T and $T_{0}$, respectively. With the temperature sensitivity coefficient defined as $K=\frac{\partial \left[L\left(T\right)-L\left(T_{0}\right)\right]}{\partial T}$, Equation (1) can be rewritten as

(2) $$T\approx T_{0}+\frac{\left[L\left(T\right)-L\left(T_{0}\right)\right]}{K}.$$

Considering the variation of reflected light intensity in unit exposure time, the corresponding temperature sensitivity coefficient $K_{t}$ is

(3) $$K_{t}=\frac{\frac{\partial \left[L\left(T\right)-L\left(T_{0}\right)\right]}{\partial t}}{\partial T}.$$

Then, when the exposure time is $t_{x}$, the corresponding temperature sensitivity coefficient$\;K_{x}=K_{t}t_{x}$.

The temperature of the LUT is expressed as follows:

(4) $$T_{s}\approx T_{0}+\frac{L_{x}\left(T_{s}\right)-L_{x}\left(T_{0}\right)}{K_{x}}=T_{0}+\frac{\Delta L_{x}}{K_{x}},$$

where $T_{S}$ is the junction temperature of the LUT, $T_{0}$ is the heat sink temperature, $L_{x}\left(T_{s}\right)$ and $L_{x}\left(T_{0}\right)$ are the reflected light intensity at the corresponding temperature when the exposure time is $t_{x}$, $K_{x}$ is the temperature sensitivity parameter (TSP), and $\Delta L_{x}$ is the intensity variation of reflected light.

For the 2D temperature distribution of the LUT, a 2D temperature-sensitive parameter matrix K(i,j) can be formulated as

(5) $$\;K\left(i,j\right)=\Delta L\left(i,j\right)/\left[T_{s}\left(i,j\right)-T_{0}\left(i,j\right)\right],$$

where (i,j) represents the arbitrary pixel point of the surface of the LUT, and K(i,j) is derived from linear fitting of the reflected light data cube as a function of heat sink temperature.

In practice, the maximum resolution of the CCD camera (1280 × 1024) was selected as the image size, and the maximum exposure time ($t_{m}$) of the corresponding camera was maintained at 1970 μs during the measurement. The number of acquired pulse cycles $=\left(t_{m}\right)/\left[\frac{pulse\;width\left(t_{n}\right)}{D}\right]\;$, where *D* refers to the duty cycle. Therefore, Equation (4) can be rewritten as

(6) $$T_{s}=T_{0}+\frac{\Delta L_{x}}{K_{x}}=T_{0}+\int_{0}^{t_{n}}\frac{\Delta L}{K_{t}t_{m}t_{n}D}dt_{n},$$

where ΔL corresponds to the total variation of reflected light intensity under $t_{m}$ exposure time, and $K_{t}$ represents the temperature sensitivity coefficient corresponding to the variation of reflected light intensity within unit exposure time.

**Figure 1:**
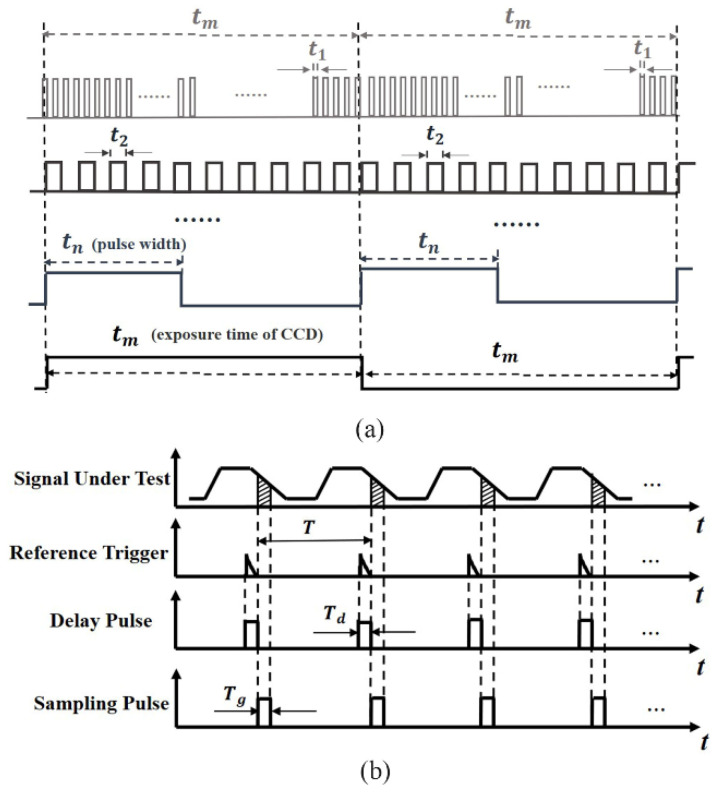
(**a**) Diagram of the pulse signal to drive the LUT (heating process); (**b**) sampling pulse setting at the falling edge (cooling process).

Figure 1 shows the pulse signal to drive the LUT in the heating process and the sampling signal in the cooling process, respectively. As shown in Figure 1a, the LUT is excited by different short-pulse widths, representing the average transient integration time. It is convenient for a signal generator to generate a nanosecond high-speed pulse signal to excite the LED chip. Because the pulse signal is too weak, the exposure time$\;t_{m}$ can be adjusted to sample multiple cycles repeatedly. A time–response curve is then obtained by sequentially changing the pulse width. The time resolution is determined by the minimum pulse width. To obtain multi-cycle image signals, a long exposure time of CCD was set to significantly increase the signal-to-noise ratio (SNR). Figure 1b shows the sampling pulse signal setting at the falling edge, i.e., the cooling process, in terms of the Boxcar principle [28]. As shown in Figure 1b, the external reference trigger from a digital delay generator was used to synchronously trigger the camera. As the pulse delay moves sequentially, signals at different stages in the cooling process can be collected until the entire cooling process is completed. The sampling pulse corresponds to the exposure time of the camera; hence, its minimum sampling time is subject to the minimum exposure time of the camera.

## 3. Experiments

**Figure 2:**
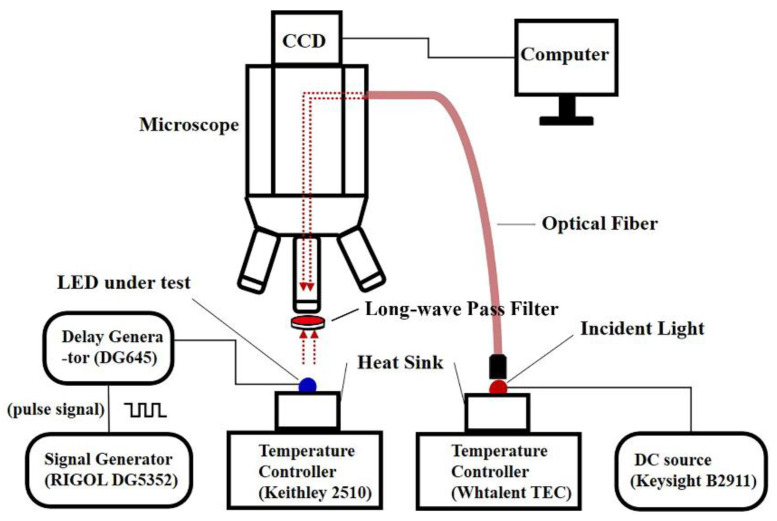
The experimental setup.

As illustrated in Figure 2, the schematic diagram for measuring the 2D transient temperature distribution of the LEDs includes the arbitrary function signal generator (RIGOL DG5352, RIGOL TECHNOLOGIES CO., LTD., Beijing, China), the CCD camera, the optical microscope with filters, the optical fiber, the incident light, the LUT, the precision source/measure unit (Keysight B2911, Keysight Technologies, Santa Rosa, CA, USA), and temperature-controlling devices (Keithley 2510 and Whtalent TEC, Keithley, Cleveland, OH, USA and Wuhan Tailunte Century Technology Co., Ltd., Wuhan, China). The incident light was provided by a high-power near-infrared chip (integrated with four 690 nm LEDs). The 408 nm (with the chip area of 1 mm × 1 mm) blue bare LED was used as the LUT. To avoid the extra excitation of photoluminescent emission from the LUT and bandgap modulation [14], the incident light with a wavelength much longer than that of the LUT was selected.

**Figure 3:**
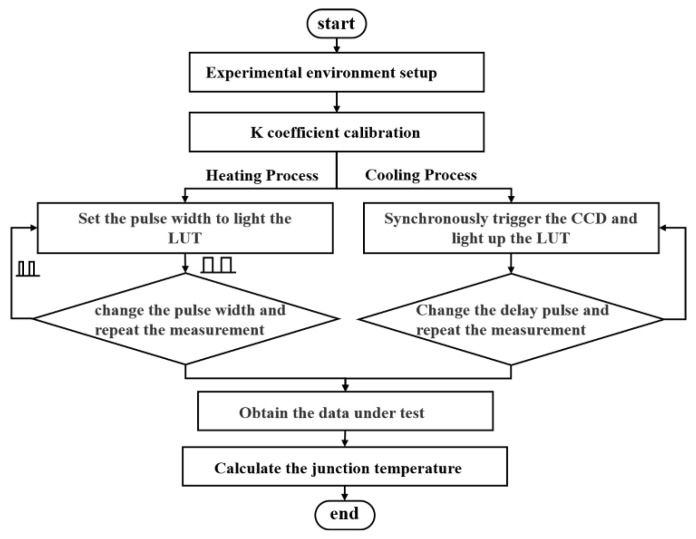
The flowchart of the experiment.

To avoid the interference of the LUT luminescence and ambient light on the experimental results, a long-wave pass filter (about 650 nm) was inserted between the LUT and the objective lens. An electrical source meter (Keysight B2911, Keysight Technologies, America) supplied direct current (DC) for the incident light source (500 mA), whose heat sink temperature was controlled at 25 °C by a temperature-controlling device (Whtalent TEC, Wuhan Tailunte Century Technology Co., Ltd., Wuhan, China). Meanwhile, an arbitrary function signal generator provided pulse signals for the LUT, the heat sink temperature of which was controlled by the temperature-controlling device (Keithley 2510, Keithley, America). The flowchart for measuring the heating the cooling processes can be found in Figure 3.

In the K coefficient calibration process, the heat sink temperature of LUT under the unlit state was adjusted from 25 °C to 50 °C with an incremental interval of 5 °C. Because the LUT was unlit, it was reasonable to use the heat sink temperature as the reference junction temperature to calibrate the K coefficient matrix.

**Figure 4:**
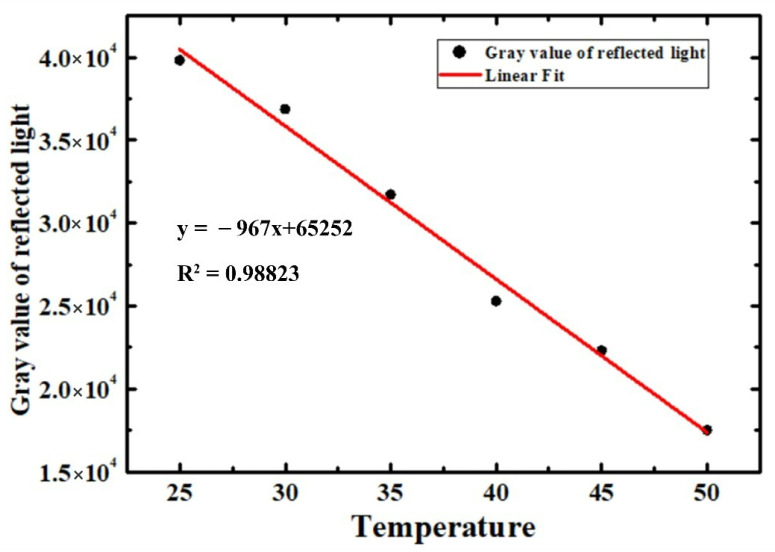
The linear fitting of the reflected light gray value as a function of the junction temperature.

**Figure 5:**
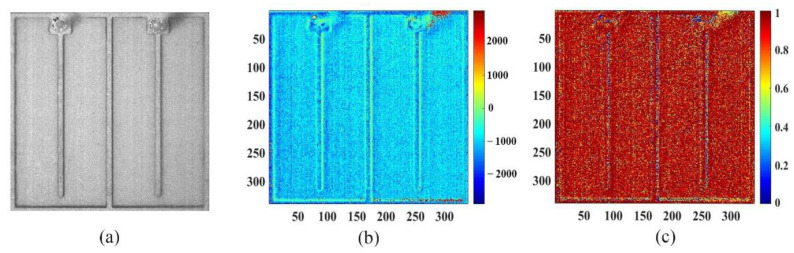
(**a**) Morphology of the LUT; (**b**) 2D temperature sensitivity parameter of the LUT; (**c**) goodness of fit.

As shown in Figure 4, the reflected light gray value of the entire LUT was selected to draw the linear fitting with the junction temperature. Meanwhile, the 2D distribution of temperature sensitivity parameters obtained by fitting and the corresponding goodness of fitting are given in Figure 5. Then, the 2D junction temperature distribution of the LUT could be derived in terms of Equation (4).

During the heating process, the LUT is excited by a short pulse with various pulse widths. The pulse width ranges from 5 ns to 10 μs with the duty cycle set to 50%.

Because the exposure time, 1970 μs, was independent of the pulse width, the number of repeated acquisitions varied according to the pulse width.

During the cooling process, a digital signal generator drove the LUT and triggered the camera simultaneously. The signal used to trigger the camera was attached to a digital delay device (DG645), which was employed to delay the signal received from the signal generator. The exposure time of the camera was set to 10 μs. The data of 100 collections were superimposed. Following each collection of data, the signal loaded on the camera was delayed by 10 μs using the digital delay generator, and the process was repeated until the entire cooling process of the LUT was collected. The two-dimensional transient junction temperature distribution of heating and cooling processes could be calculated using Equation (6).

## 4. Results and Discussion

**Figure 6:**
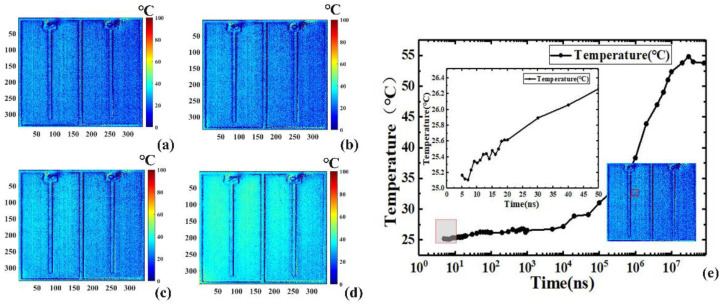
Transient 2D temperature distribution of the LUT driven by various pulse widths in the heating process of (**a**) 10 ns, (**b**) 500 ns, (**c**) 100 μs, and (**d**) 1 ms; (**e**) the time–response curves in logarithmic coordinates of average transient junction temperature in the marked red square box.

**Figure 7:**
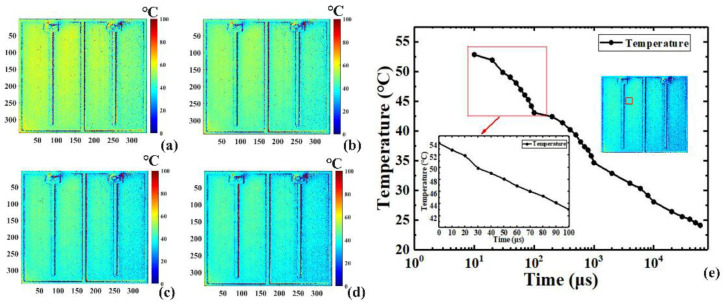
Transient 2D temperature distribution of the LUT driven by various pulse widths in the cooling process of (**a**) 0 μs, (**b**) 50 μs, (**c**) 100 μs, and (**d**) 200 μs; (**e**) the time–response curves in logarithmic coordinates of the average transient junction temperature of the marked red square box.

Figure 6 and Figure 7 show the two-dimensional transient junction temperature distribution of the LUT at different timepoints in the heating and the cooling processes, with ns- and μs-scale resolutions, respectively Figure 6a–d show the transient 2D temperature distributions of the LUT driven by various pulse widths in the heating process, while Figure 6e shows the time response curves in logarithmic coordinates of the average transient junction temperature of a 40 × 40 pixel region marked with the red square box indicated in the inset of Figure 6e.

The TSP and the intensity of reflected light in a single period were calculated by loading the periodic pulse signal to the LUT. The minimum 5 ns resolution is beyond the time resolution of an ordinary camera. Because the signal acquisition time was extremely short at first, the 2D temperature distribution variation was not obvious, as illustrated in the inset of Figure 6e, the temperature only increased about 1.2 °C from 5 ns to 50 ns. The temperature began to increase at 10 µs and reached a peak around 10 ms. Figure 7 shows the time response of the cooling process. Similar to the heating process, the same region of 40 × 40 pixels marked with a red square box was also used to illustrate the average transient temperature curve in logarithmic coordinates, as indicated in the inset of Figure 7e. Subject to the minimum exposure time of the CCD camera, the temporal resolution in the cooling process was on the μs scale.

To verify the accuracy of the experimental results, we measured the steady-state average temperature of the LUT using a micro-thermocouple three times, achieving results of 55.1 °C, 56.7 °C, and 56.8 °C with an average of 56.2 °C. This was compared with the average junction temperature, 53.8 °C, 54.8 °C, 54.0 °C, and 53.8 °C at 20 ms, 30 ms, 40 ms, and 80 ms obtained in the transient heating experiment, with errors of 4.4%, 2.4%, 4.0%, and 4.3%, respectively, and an average error of 3.8%, indicating the high consistency and accuracy of the proposed method.

**Figure 8:**
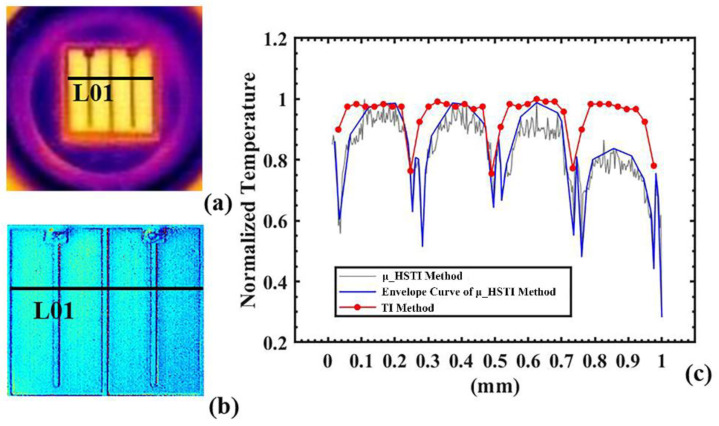
(**a**) TI method; (**b**) µ_HSTI method; (**c**) normalized temperature distribution of the blue LUT, measured using the µ_HSTI method (the black line) and the TI method (the red line).

As illustrated in Figure 8, the spatial temperature distribution given by the μ_HSTI was compared with the infrared thermal imaging method (TI method) [29]. The trend of the proposed method was consistent with that of the TI method, whereas more details, i.e., higher spatial resolution, were revealed in the proposed method. Considering that the incident light beam’s homogeneity impacts the intensity of reflected light in different areas, the SNR can be further enhanced to reduce the fluctuation in temperature distribution by increasing either the incident light intensity or the homogeneity of the incident light. A higher reflectivity of electrodes of the LUT resulted in more significant temperature fluctuation at the electrodes, as illustrated in Figure 8c, where valley points of the curve correspond to the electrodes. The temperature trends at electrodes were consistent with those measured by the TI method.

## 5. Conclusions

In summary, by applying a periodic short-pulse signal to drive the LED chip, we successfully overcame the ordinary camera’s time resolution limitation, obtaining the two-dimensional transient junction temperature distribution in the heating process with a time resolution up to nanosecond scale while maintaining the spatial accuracy. The temporal resolution of our method was determined by the pulse width of the signal generator. In the cooling process, the Boxcar gated integration technique was adopted to sequentially acquire the two-dimensional transient cooling temperature distribution with the time resolution subject to the minimum exposure time of the camera, 1 μs in this case. A comparative experiment with the thermal imaging method confirmed the consistency in the temperature trend. The method we proposed in this work is also applicable to other semiconductors with a PN junction that do not emit light, as long as the semiconductors can be driven by short-pulse signals.

## Data Availability

Not applicable.
